# Secondary Brain Injury by Oxidative Stress After Cerebral Hemorrhage: Recent Advances

**DOI:** 10.3389/fncel.2022.853589

**Published:** 2022-06-23

**Authors:** Linqian Shao, Sichao Chen, Li Ma

**Affiliations:** Department of Neurosurgery, Sir Run Run Shaw Hospital, Zhejiang University School of Medicine, Hangzhou, China

**Keywords:** cerebral hemorrhage, oxidative stress, research progress, Nrf2, heme oxygenase

## Abstract

Intracerebral hemorrhage (ICH) is a clinical syndrome in which blood accumulates in the brain parenchyma because of a nontraumatic rupture of a blood vessel. Because of its high morbidity and mortality rate and the lack of effective therapy, the treatment of ICH has become a hot research topic. Meanwhile, Oxidative stress is one of the main causes of secondary brain injury(SBI) after ICH. Therefore, there is a need for an in-depth study of oxidative stress after ICH. This review will discuss the pathway and effects of oxidative stress after ICH and its relationship with inflammation and autophagy, as well as the current antioxidant therapy for ICH with a view to deriving better therapeutic tools or targets for ICH.

## Intracerebral Hemorrhage

Intracerebral hemorrhage (ICH) is the second most common type of stroke in the world, accounting for 10–15% of all strokes (Feigin et al., 2009; Steiner et al., 2014; Kim and Bae, 2017). The early brain damage from ICH is mainly caused by the mass effect from the formation of a hematoma. However, the patient's clinical symptoms are not alleviated or improved when bleeding stops. Therefore, the study of the mechanism of secondary injury occurring after ICH has led to a more extensive and in-depth exploration and discussion among researchers (Babu et al., 2012). Secondary brain damage after ICH mainly comes from processes such as oxidative stress and inflammation (Aronowski and Zhao, 2011). After ICH occurs, blood components quickly migrate from the vessel, including red blood cells, white blood cells, macrophages, and hemoglobin. Additionally, the divalent iron ions produced during hemoglobin cleavage can promote the formation of free radicals, leading to the occurrence of oxidative stress (Xiong et al., 2014). The body's oxidative stress response can then activate nuclear factor-κB (NF-κB), a key regulator of inflammation, inducing the expression of proinflammatory cytokines (Khaper et al., 2010; Hu W. et al., 2015). Proinflammatory cytokines may also participate in promoting the generation of free radicals, leading to a malignant positive feedback loop (Duan et al., 2016).

## Pathophysiological Mechanisms of ICH

The pathophysiology of ICH is complex and includes the totality of the brain injury, from early hematoma formation and expansion to SBI from oxidative stress, inflammatory response, mitochondrial dysfunction, and cell death. In contrast, oxidative stress is the main source of SBI in ICH.

### Mitochondrial Dysfunction and Cell Death

As a structure playing an important role in the redox homeostasis of the whole cell (Georgieva et al., 2017), mitochondria are inextricably linked to the pathophysiological mechanisms associated with SBI in ICH (Chen W. et al., 2020). It has been shown that the incidence of mitochondrial dysfunction in the hematoma area is significantly higher during SBI and that mitochondria with normal functional activity play a significant role in the maintenance of neuronal survival (Kim-Han et al., 2006; Diao et al., 2020; Chen et al., 2021), indicating that mitochondrial dysfunction is closely related to SBI. Cell death after ICH is mainly divided into two categories: apoptosis and necrosis. Numerous preliminary and clinical studies have shown that apoptosis is involved in the physiopathological process of ICH (Chu et al., 2014; Salihu et al., 2016; Zille et al., 2017). After ICH, substances such as inflammatory cytokines that are released in response to oxidative stress activate cysteinases. They injure or kill cells through cysteinase-dependent or independent pathways, allowing apoptosis to occur. The mechanical compression of adjacent tissues by the hematoma and the accumulation of excess glutamate after ICH cause activation of N-methyl-d-aspartate (NMDA) receptors. large influx of Ca^2+^, intracellular Ca^2+^ overload, and consequent mitochondrial dysfunction occur. Eventually, cellular necrosis occurs due to insufficient ATP production by mitochondria (Zhang et al., 2022).

### Oxidative Stress and ROS

Oxidative stress is a state in which oxidant and antioxidant effects in the body are out of balance. Under this state, the body produces excessive free radicals, which cause serious oxidative damage to cells, eventually damaging cell vitality or bringing about cell apoptosis (Sinha et al., 2013). The main component of free radicals in organisms is active oxygen, which includes hydroxyl radicals (·OH), superoxide anions (O^·2−^), hydrogen peroxide (H_2_*O*_2_), and other substances. These are normally found in low concentrations in the body and participate in redox reactions. In low concentrations, part of the active oxygen can also participate in the regulation of the signal transduction pathway, as the role active oxygen plays in insulin signal transduction. In cases with excessively high concentrations, active oxygen may cause lipid oxidation or the oxidation of quality proteins and DNA, and which ultimately, may promote cell apoptosis or death (Steinbrenner and Sies, 2009; Qu et al., 2016). Meanwhile, reactive oxygen species (ROS) is one of the main influencing factors for SBI in ICH (Duan et al., 2016; Qu et al., 2016), and the generation and accumulation of excessive ROS can cause adverse outcomes, such as macromolecular damage, impaired cell signaling, cell death, and tissue damage, which can lead to further SBI (Forrester et al., 2018). In a normal physiological state, ROS would be in a dynamic equilibrium in the body (Turrens and Boveris, 1980), whereas after the occurrence of ICH, a large amount of ROS is generated, and the accumulation of ROS in excess leads to oxidative stress, aggravating SBI (Duan et al., 2016). In addition to this, excess ROS will cause damage to the normal function of mitochondria, producing mitochondrial dysfunction and leading to the generation of additional ROS, which leads to cascading cell damage. This makes the degree of SBI after ICH worse (Qu et al., 2016).

## Oxidative Stress After ICH

Oxidative stress plays an essential role in the development of ICH, and there is a causal relationship between the excess increase in free radicals and the damage caused by ICH (Aronowski and Zhao, 2011). After the occurrence of ICH, there is a massive increase in ROS, an imbalance between oxidation and antioxidation occurs. This results in oxidative stress, which leads to damaged brain cells and the destruction of the blood–brain barrier (BBB) (Qu et al., 2016).

### Sources of Free Radicals After ICH

**Figure 1:**
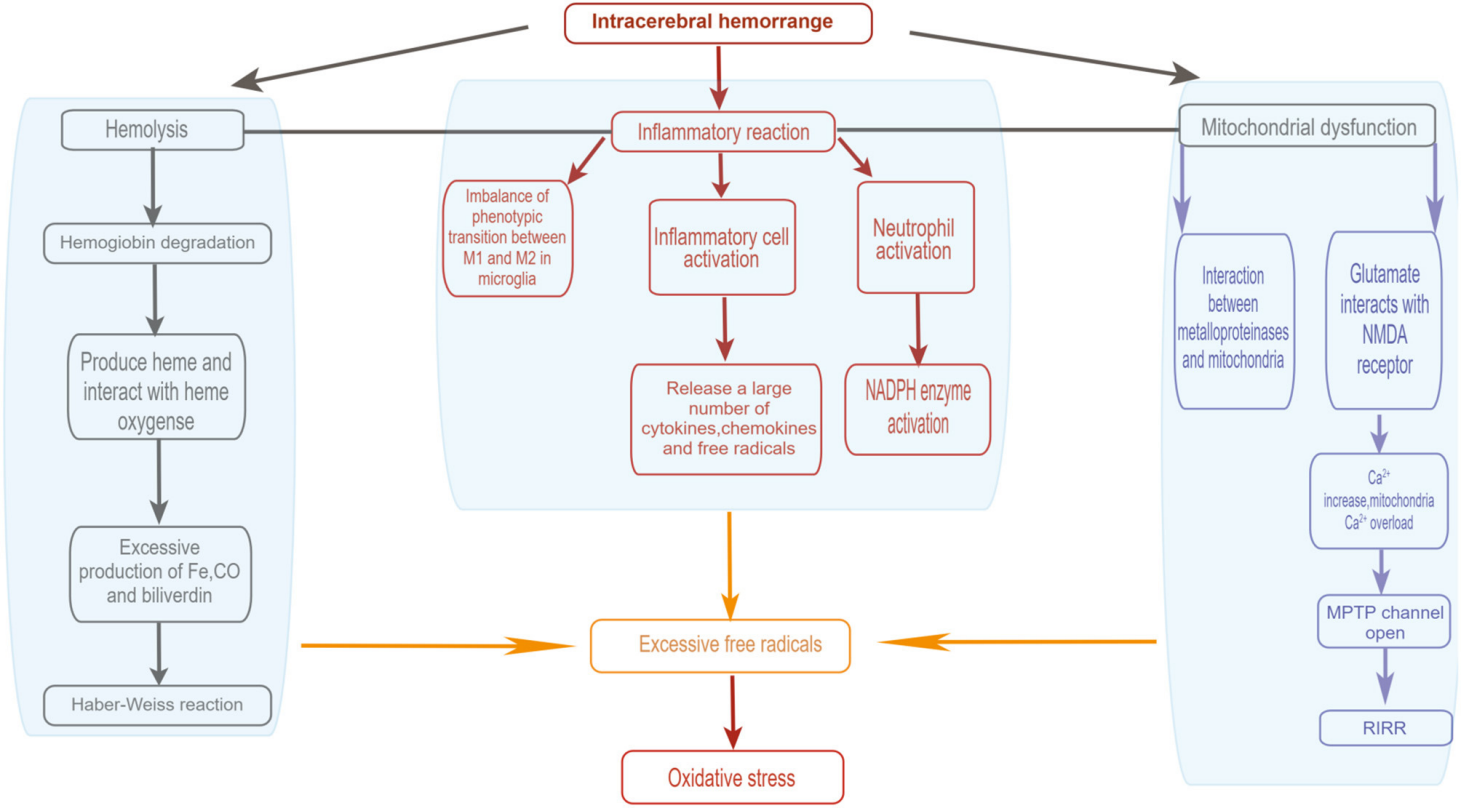
Sources of oxidative' stress after intracerebral hemorrhage. (1) Activated inflammtory cells include leukocytes, macrophages and microglia. (2) After ICH, glutamate is released into the bloodstream, and glutamate interacts with NMDA receptors, leading to an increase in Ca^2+^ concentration and thus Ca^2+^ overload in the mitochondria, which opens the mitochondrial permeability transition pore (MPTP) and disrupts the mitochondrial respiratory chain, resulting in the release of reactive oxygen species. (3) RIRR, ROS-induced ROS release. By Figdraw (www.figdraw.com).

There are many sources of free radicals after ICH (Figure 1). After bleeding, blood cells dissolve and hemoglobin is released. Simultaneously, the heme in the degradation product of hemoglobin is decomposed into iron, carbon monoxide, and biliverdin under the action of heme oxygenase (Maines, 1997; Wang and Doré, 2007b). The excess iron produced in extracellular space will have harmful effects. The Haber-Weiss reaction will occur under the catalysis of free iron, resulting in enhanced damage to neurons. At the same time, highly reactive toxic hydroxyl radicals are produced in the process. With this, oxidative stress and cell death will occur, resulting in lipid peroxidation, and excitotoxicity will also be enhanced (Regan and Panter, 1996; Goldstein et al., 2003). Moreover, inflammation is another major source of ROS after cerebral hemorrhage. When ICH occurs, blood components will penetrate the injured site. The inflammatory reaction occurs rapidly, leading to the activetion of various inflammatory cells (Wang and Tsirka, 2005a; Wang and Doré, 2007a). Meanwhile, a large number of cytokines, chemokines, and free radicals are released by activated inflammatory cells such as microglia (Aronowski and Hall, 2005; Wang and Tsirka, 2005a; Wang and Doré, 2007a; Gao et al., 2008). Microglia produce two different phenotypes: the M1 phenotype, which is activated by the classical pathway, and the M2 phenotype, which is activated alternately. Microglia of the M1 phenotype can be considered as pro-inflammatory cells. After ICH, microglia of the M1 phenotype secrete large amounts of ROS and pro-inflammatory factors. Microglia of the M2 phenotype are currently considered as protective cells, secreting anti-inflammatory factors and upregulating neuroprotective factors. Study showed that most of the newly recruited microglia at the injury site were M2 phenotype, while M1 phenotype dominated about 1 week after injury. This phenotype shift from M2-dominant to M1-dominant may result from a M2-to-M1 conversion within activated microglia. The imbalance in the number of the two phenotypes produced leads to the accumulation of ROS and a high production of pro-inflammatory factors (Hu W. et al., 2015). Moreover, the activation of neutrophils also result in the activation of NADPH Oxidase (Nox) and production of ROS (Joice et al., 2009). In addition to the main sources mentioned above, another source of ROS after ICH is mitochondrial dysfunction (Kim-Han et al., 2006; Swanson, 2006). The excess iron produced after ICH will induce mitochondrial dysfunction and produce oxidative damage. Mantle et al. demonstrated that impaired mitochondrial function leads to a significant increase in ROS (Mantle et al., 2001). At the same time, the release of inflammatory mediators and metalloproteinases also mediates oxidative damage (Gong et al., 2000; Alvarez-Sabín et al., 2004). Furthermore, after ICH, glutamate is released into the blood and interacts with N-methyl-D-aspartate receptors, leading to Ca^2+^ overload in the mitochondria (Sharp et al., 2008). Activated α-amino-3-hydroxy-5-methyl-4-isoxazole propionic acid receptors may further promote mitochondrial Ca^2+^ overload (Joshi et al., 2015). The loading of mitochondrial Ca^2+^ reduces its transmembrane potential and opens the mitochondrial permeability transition pore (MPTP), which destroys the mitochondria and mitochondrial respiratory chain. This then leads to the release of ROS (Mracsko and Veltkamp, 2014). Besides, the activation of MPTP changes the mitochondria. The internal redox environment promotes the release of active oxygen by positive feedback, forming the “active oxygen-induced active oxygen release” (RIRR) (Zorov et al., 2014).

### Effects of Oxidative Stress After ICH

The excessive generation of free radicals is one of the main attributer in the formation of brain damage after ICH. After the occurrence of ICH, a major number of free radicals are produced, while excessive consumption of superoxide dismutase (SOD) occurs at the same time. It result in the occurrence of lipid peroxidation (Chi et al., 2014), which can by oxygen free radicals causing cell damage. Brain is sensible to oxygen free radicals with rich lipid content. Lipids are subject to peroxidation in the presence of large amounts of oxygen free radicals, causing damage to cells and further leading to membrane damage. Damage to the membrane leads to its increased permeability and increased influx of Ca^2+^(Toda et al., 2009). The activity of membrane proteins such as Na ^+^/Ca^2+^ exchangers is inhibited by cross-linking and polymerisation of membrane lipids due to lipid peroxidation (Eigel et al., 2007), further increasing the concentration of intracellular calcium. The absorbed calcium combines with phosphate to form insoluble calcium phosphate, which affects the process of mitochondrial oxidative phosphorylation and reduces the production of ATP (Li et al., 2014). Because of the increase in Ca^2+^ concentration, phospholipase is activated, and membrane phosphorylation occurs in cell and organelle membranes (Chrissobolis et al., 2011; Gu et al., 2011). Free radicals cause a large amount of damage to neuron. It not only damages the cell membrane, but also may lead to disruption of cellular DNA (Mantle et al., 2001). Free radicals react with the components of DNA molecules, and this can cause damage to the purine and pyrimidine bases and deoxyribose backbone (Dizdaroglu et al., 2002). The DNA damage may cause transcriptional arrest, signal transduction pathway induction, replication errors, and genome instability, leading to neuronal apoptosis (Marnett, 2000; Cooke et al., 2003)_._ Numerous studies have shown that free radicals are closely to pathophysiological process to ICH. Malondialdehyde (MDA) is a product of free radicals, whose concentration level is often used to evaluate the protein oxidation degree of damage. Studies have shown that the level of MDA increases after ICH. The increased levels of MDA can induce the apoptosis of neuron and glial, indicating that an oxidative stress response promotes the occurrence of ICH-induced brain damage (Wagner et al., 2002; Han et al., 2008).

### Endogenous Antioxidant Mechanisms in ICH

#### Antioxidant Mechanism of Heme Oxygenase

HO (heme oxygenase system) is composed of two definite forms: oxidative stress-inducing protein HO-1 and constitutive isoenzyme HO-2.HO-1 is produced under the induction of microglia/macrophages. While HO-2 is usually dominant in the expression of neurons and is the main cause behind the activity of HO in the brain (Muñoz-Sánchez and Chánez-Cárdenas, 2014). As a part of hemoblood degradation, microparticles constitutes a HO system. Under normal circumstances, the HO system regulates hemoglobin levels and protects cells from the harmful effects of intracellular free hemoglobin (Yoshida and Migita, 2000; Kikuchi et al., 2005; Kumar and Bandyopadhyay, 2005). In fact, after the occurrence of ICH, the lysis of red blood cells within the hematoma leads to release of hemoglobin, which is later broken down into heme. The toxic effect of heme causes secondary damage in ICH. Serum proteins and albumin can bind to hemoglobin and prevent toxic effects. However the amount of hemoglobin released from the hematoma is too large, the natural defense mechanism mentioned above cannot completely remove the hemoglobin released from the hematoma (Robinson et al., 2009). After ICH, HO promotes the decomposition of heme to produce carbon monoxide, iron, and other substances (Maines, 1997; Wang and Doré, 2007b), while excess iron is a component of SBI after ICH (Xi et al., 2006; Keep et al., 2012; Jin et al., 2013). However, the effects of these two types of HO after ICH are variable, they will be affected by the type of model and type of cells involved, producing different results (Chen-Roetling et al., 2015). It was revealed that in a collagenase-induced ICH model, although HO-1 induced early brain damage in ICH, neurological function improved over time in WT mice, while it remained unchanged in HO-1-deficient mice. This is because activated microglia/macrophages are a key factor in the rapid clearance of dying cells and elimination of haematomas, as well as an important supplier of neuroprotective molecules. The enhanced HO-1 activity found experimentally may be necessary for the optimal function of ICH-activated microglia/macrophages. This fact may confirm the protective effect of HO-1 induction in the WT group during ICH recovery (Wang and Doré, 2007b). In a therapeutic study of ICH with sulforaphane, HO-1 was upregulated in response to sulforaphane, resulting in a significant reduction in brain damage, and there was a significant correlation between the two (Zhao et al., 2007). At the same time, the role of HO-2 is more ambiguous. *In vitro* studies showed that knocking out HO-2 can play a role in resisting hemoglobin or heme damage and protecting mouse neurons (Rogers et al., 2003; Regan et al., 2004). However, collagenase injection to ICH mice model, HO-2 knockout, it's had little of any neuronal protection (Wang et al., 2006; Chen-Roetling et al., 2013). Therefore, the antioxidant effect of the HO system after ICH needs to be viewed dialectically. More in-depth research is needed to see if it can play a more beneficial role in the treatment of ICH.

#### Antioxidant Mechanism of Superoxide Dismutase

Superoxide dismutase (SOD) is an important antioxidant enzyme that plays a role in maintaining $O_{2}^{-}$ homeostasis in the antioxidant defense system (Batinic-Haberle et al., 2014). SOD as a system for maintaining homeostasis of $O_{2}^{-}$ levels, is composed of three isoforms. Cu/ZnSOD (SOD1) in the cytoplasm, MnSOD (SOD2) in the mitochondria, and Cu/ZnSOD (SOD3) located outside the cell (Fukai and Ushio-Fukai, 2011). Superoxide radicals are first detoxified in the cytoplasm by the action of SOD1 and SOD2, followed by further detoxificatial into water (Valko et al., 2006).

SOD1 deficiency increases superoxide and produces vascular dysfunction in large arteries and microvessels, exacerbates vascular dysfunction produced by angiotensin II, and increases matrix metalloproteinase 9 (MMP-9) expression and activation (Gasche et al., 2001; Didion et al., 2002, 2005). In contrast, SOD1 overexpression will reduce oxidative stress, attenuate the induction and activation of MMP-9, and prevent the development of vascular dysfunction (Morita-Fujimura et al., 2000; Didion et al., 2005). In an ICH model experiment, the work of Yoshinobu et al. verified the inference that overexpression of SOD1 reduce superoxide production and decrease secondary damage in ICH (Wakisaka et al., 2010).

Brain endothelial cells contain a greater number of mitochondria. SOD2 is mainly found in mitochondria. Schroeter et al. design two types of astrocytes and endothelial cells co-culture models. The results showed that the activity of SOD2 in endothelial cells increased after incubation with astrocytes for 48 h. It indicates astrocytes induce SOD2 in brain endothelial cells by either direct contact or exchange of soluble factors. Astrocytes induce BBB properties in brain endothelial cells, and high SOD activity is a prerequisite for normal BBB function (Schroeter et al., 2001). Consequently, SOD2 may have an important share in the protective mechanism of the cerebrovascular system. In the study by Faraci et al. SOD2 reduced brain damage by reducing superoxide production and protecting cells from oxidative stress (Faraci et al., 1985).

In ICH animal experiments, the levels of both SOD1 and SOD2 content in the ipsilateral striatum were reduced and the free radical scavenging system was impaired, leading to the development of neurological impairment in rats, which reflects the development of oxidative damage in the brain after ICH (Wu et al., 2002). In contrast, in clinical studies, the antioxidant system was impaired after the onset of ICH. The impaired free radical scavenging system after ICH correlates with the decrease in SOD levels (Aygul et al., 2005; Chen et al., 2014). In conclusion, the antioxidant mechanism of SOD has important research significance in SBI after ICH.

#### Antioxidant Mechanism of Nrf2

**Figure 2:**
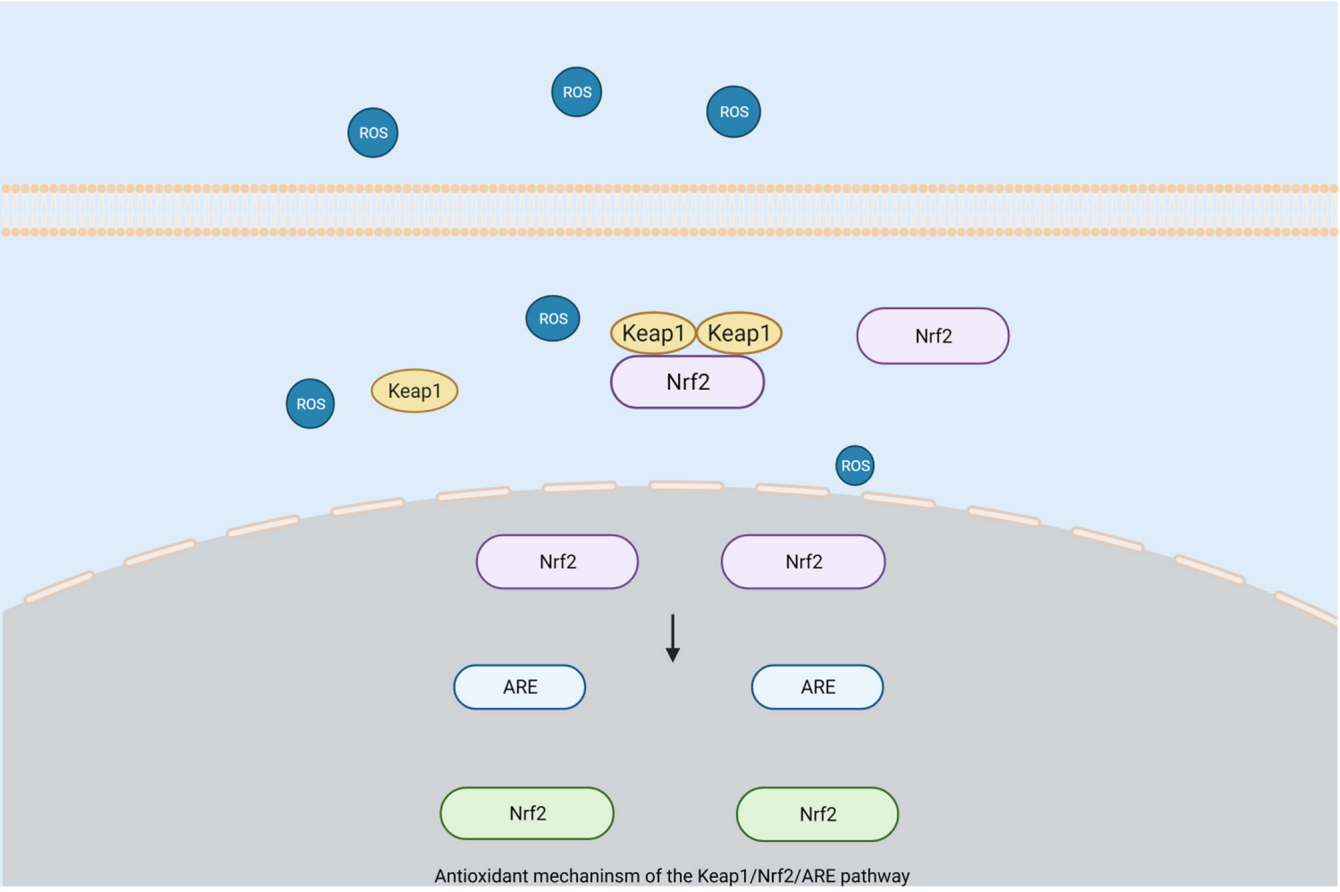
Antioxidant mechanism of the Keap1/Nrf2/ARE pathway. When Nrf2 is exposed to reactive oxygen species, it will dissociate from Keap1 and translocate to the nucleus, while activating antioxidant response elements (ARE) that mediate cell survival to drive the expression of Nrf2 target genes for protective purposes. (Created with BioRender.com).

Nrf2 belongs to one of the basic regions of the leucine zipper proteins and is the main genomic regulator of the cellular antioxidant defense system. It can participate in the regulation of the antioxidant processes of HO and SOD (Zhao and Aronowski, 2013). Activated Nrf2 is released from Keap1 to increase cell protection and the expression levels of antioxidant target genes so that the cell's defense against oxidative stress is enhanced (van Muiswinkel and Kuiperij, 2005; Kensler et al., 2007). Studies have shown that the activation of Nrf2 plays an important protective role in the occurrence of oxidative stress after an ICH. In mice lacking Nrf2, the damage caused by oxidative stress after ICH is more obvious (Zhao et al., 2007). ROS released by oxidative stress can reduce the damage caused after ICH by activating the Keap1/Nrf2/ARE pathway (Gasche et al., 2001; Wang et al., 2007; Zhao et al., 2007) (Figure 2). As the main regulator, the Keap1-Nrf2 pathway can protect cells against endogenous and exogenous stress caused by ROS and electrophiles (Kansanen et al., 2012). Keap1 can bind to Nrf2 and promote its degradation through the ubiquitin proteasome pathway, which has a negative regulatory effect on Nrf2. When Nrf2 is exposed to ROS, it dissociates from Keap1 and transfers to the nucleus. Simultaneously, it activates the antioxidant response element (ARE) that mediates cell survival so as to drive the expression of the target gene of Nrf2 (Kansanen et al., 2013; Qaisiya et al., 2014). Experiments showed that Nrf2 increased significantly at 22 h after the occurrence of ICH, while Keap1 showed a corresponding decrease (Wada et al., 1970). Hence, Keap1 is inhibited and Nrf2 is activated after the occurrence of ICH(Zhao et al., 2007). In addition, results have shown that the degree of brain damage in mice with Nrf2 knockout is more serious, which can indicate a neuroprotective effect of Nrf2 after ICH (Wang et al., 2007; Zhao et al., 2007).

Haptoglobin (Hp) is a glycoprotein that can form a highly stable Hb–Hp complex with free Hb (Wada et al., 1970). Free Hb produced by oxidative stress after ICH may cause strong lipid peroxidation, oxidative DNA damage, and neuronal death (Sadrzadeh et al., 1984; Keep et al., 1998; Wang et al., 2002), so the toxic effect of Hb may be prevented by Hp's neutralization of Hb. It has been shown that after ICH, the levels of Hp in the plasma of animals treated with Nrf2 activators increased significantly, the activation of Nrf2 may be related to an increase of Hp levels (Zhao and Aronowski, 2013). Nrf2 may achieve a protective effect of brain injury after ICH by regulating the level of Hp content in the brain.

At the same time, since excess heme produced after ICH can also have toxic effects, and hemopexin is a protein that can bind to heme with a strong affinity (Nikkil et al., 1991; Tolosano and Altruda, 2002). Reduce of free heme can be achieved by binding hemopexin and heme, thus alleviating the oxidative damage and toxic effects caused by excess heme. There has been evidence that the activation of Nrf2 can enhance the expression of hemopexin (Shen et al., 2006; Kristiansson et al., 2007), which subsequently has a positive effect on clearing out heme (Zhao and Aronowski, 2013).

#### Other Pathway That Have Antioxidant Effect

Phosphatidylinositol 3-kinase (PI3-K) is an enzyme with serine/threonine protein kinase activity that is involved in immune cell activation signaling and activation. Akt is a widely expressed cytoplasmic serine-threonine kinase that plays an important role in cell survival and apoptosis. The PI3K/Akt pathway is a signaling pathway that is important for neuronal survival regulation (Dudek et al., 1997). Previous studies have shown that PI3K/Akt is a neuroprotective signaling possesses antineuroinflammatory, antioxidative stress, and antiapoptotic properties (Tu et al., 2016; Lv et al., 2017). Therefore, researchers have investigated the role of this pathway in SBI after ICH, showing that activation of the PI3K/Akt pathway by different targets after the occurrence of ICH leads to positive results in terms of the attenuation of neuroinflammation and oxidative stress. It demonstrates the neuroprotective role of the PI3K/Akt pathway in SBI after ICH (Zhao et al., 2019; Chen S. et al., 2020).

## Association Between Oxidative Stress and Other Responses After ICH

### Interaction Between Oxidative Stress and Autophagy After ICH

Autophagy is a degradation process that removes damaged organelles or misfolded proteins. This process can help maintain cell homeostasis and eliminate the damage caused by oxidative stress or protein toxic stress (Jiang et al., 2015). Experimental results have shown that the overload of iron produced under oxidative stress may play a key role in inducing autophagy caused by ICH (He et al., 2008). Previous studies have shown that autophagy act as a negative feedback in the pathological process of ICH (He et al., 2008; Lee et al., 2009; Hu et al., 2011; Wang et al., 2012; Liu et al., 2014). ROS and other free radical substances generated by oxidative stress may induce autophagy. On the other hand, the corresponding stress products can be cleared by autophagy to reduce brain damage (Filomeni et al., 2015; Ismail et al., 2020). The protein p62 binds to ubiquitinated protein in the process of autophagy and transfers it to the autophagosome, which promotes selective protein degradation (Jiang et al., 2015). Phagocytosis regulated by the p62 pathway has a corresponding protective effect on oxidative stress response to brain injury, demonstrating the regulatory effect of autophagy on oxidative stress response after ICH (Rubio et al., 2014). In addition, a series of findings suggest that autophagy can regulate ROS content through the p62 pathway, the chaperone-mediated autophagic pathway, and the mitochondrial pathway. These findings provide a more in-depth theoretical basis for the pathogenesis of cerebral hemorrhage, but due to the diversity of its internal molecular regulatory mechanisms, its specific results need to be further investigated and discussed (Tan et al., 2015). Another study established a cellular module associated with ICH and found that this module is enriched in protein ubiquitination pathways that regulate neuroinflammation and autophagy and enhance the oxidative effects associated with the Nrf2 pathway. It also modulates microglia function and improves the antioxidant capacity of the body after ICH, which reinterprets the relationship between autophagy and oxidative stress after ICH from another perspective (Durocher et al., 2019).

#### Interaction Between Oxidative Stress and Inflammatory Response After ICH

A growing body of experimental research has shown that inflammation plays an increasingly important role in the pathophysiology of SBI after ICH. There is a close interaction between inflammatory reaction and post-ICH oxidative stress. The oxidative stress reaction that occurs after ICH can trigger an inflammatory reaction, which subsequently can cause corresponding brain damage through the oxidative stress pathway (Mracsko and Veltkamp, 2014). For example, pro-inflammatory cytokines, such as tumor necrosis factor and interleukin 1, is expressed under the induction of free radical substances. Free radical substances produced by oxidative stress play an important role in the induction and occurrence of inflammatory reactions (Khaper et al., 2010; Hu X. et al., 2015), while pro-inflammatory cytokines can also produce free radicals, which promote the occurrence and progress of oxidative stress (Khaper et al., 2010).

The inflammatory response after ICH leads to the activation or infiltration of inflammatory cells and the mass production of inflammatory factors and chemokines. Excessive inflammatory factors activate the NF-κB signaling pathway. It inhibits the Nrf2 pathway, which leads to oxidative stress (Aronowski and Zhao, 2011; Chaudhary et al., 2013; Zhou et al., 2014; Saha et al., 2020). At the same time, after ICH, activated inflammatory cells not only produce a large number of inflammatory factors but also promote the production of ROS and other free radical substances (Matsuo et al., 1995; Yang et al., 2015). In addition, in the inflammatory response, microglia with unbalanced phenotype transfer can cause a large amount of ROS and inflammatory factors to be released during the process of phenotypic conversion (Hu W. et al., 2015). *In vitro* experiments have also shown that microglia can induce the production of ROS (Cui et al., 2015; Yang et al., 2015), in an animal simulation experiment of ICH, when microglia were suppressed, the production of ROS and brain damage were both reduced. It demonstrates the important effect of microglia on the oxidative stress response after ICH (Wang and Tsirka, 2005b). Moreover, while Nrf2 reduces oxidative damage through the Keap1/Nrf2/ARE pathway, it also protects against inflammatory responses through the Nrf2/ARE pathway. There is a common pathway between these two (Chen et al., 2006; Thimmulappa et al., 2006). This may be achieved by inhibiting NF-κB (Thimmulappa et al., 2006), an important regulator of many pro-inflammatory genes, to achieve anti-inflammatory protection, and NF-κB regulators also play an important role in protection from oxidative stress. There is a clear and significant relationship between oxidative stress and inflammation after ICH, which provides a great reference point for further study of the injury mechanism after ICH.

## Clinical Impact of Oxidative Stress In and Treatment of ICH

### Clinical Impact of Oxidative Stress in ICH

There are a variety of markers that can reflect the level of oxidation after ICH. Substances such as malondialdehyde(MDA), SOD and glutathione-mercapto peroxidase (GSH-Px) are commonly used to monitor the course of the oxidative stress response after ICH. Researchers have studied the impact of OS in plasma and cerebrospinal fluid (CSF) of patients with ICH on their clinical outcomes. The results showed that elevated MDA in CSF and total antioxidant status in plasma were associated with harmful outcomes, while higher plasma SOD and GSH-Px were associated with favorable outcomes in ICH (Masomi-Bornwasser et al., 2021). This reflects the predictive power of oxidative markers for ICH outcomes. Also, data suggest that leukocyte 8-hydroxy-2'-deoxyguanosine (8-OHdG) levels are higher in ICH patients than in healthy subjects (Chen et al., 2011). Since guanine is the most easily oxidized of the five nucleobases, and 8-OHdG is its oxidation product. Therefore, in response to this finding, some investigators recently analyzed serum oxidized guanine levels in patients with ICH and obtained its correlation with mortality, demonstrating that 8-OHdG could be a new independent predictor of ICH outcome (Lorente et al., 2020). Myeloperoxidase (MPO) is one of the common markers of oxidative stress, and thus the changes in MPO after ICH were clinically studied. The results showed that elevated serum MPO concentrations in ICH patients were associated with increased oxidative stress and correlated with ICH prognosis, suggesting that serum MPO levels may be one of the useful biomarkers for determining the prognosis of ICH (Zheng G. R. et al., 2018). In summary, it can be concluded that oxidative stress and its markers are of great significance in the clinical study of ICH.

### Antioxidant Therapy After ICH

Since oxidative stress damage after ICH is caused by excessive ROS and other free radical substances, antioxidant treatment after ICH can be divided into the following directions (Table 1):

**Table 1:** Antioxidant therapy for cerebral hemorrhage.

**Treatment**	**Mechanism of action**	**Research progress**	**Whether it has been tested in clinical trials and/or animal models**	**Reference**
MIS surgical treatment	Reduces the excessive production of free radicals such as ROS in the body by expelling blood, thus reducing oxidative damage	In animal studies, neurological function was better protected in animals using MIS alone or in combination with other therapies	Has been tested in clinical trials	Wu et al., 2011; Liu et al., 2016
Desferrioxamine mesylate (DFO) treatment	Binds to excess iron produced after brain hemorrhage, thereby reducing free radical production and mitigating oxidative damage	Preliminary results from phase II trial show that DFO reduces perihematoma edema	Has been tested in clinical trials	Selim et al., 2011
Selenium nanocomposite therapy	Blocking excessive production of intracellular reactive oxygen species, thus reducing oxidative damage after cerebral hemorrhage	Good efficacy for oxidative damage after cerebral hemorrhage was achieved in animal experiments	Has been tested in animal models	Yeatts et al., 2013
Nxy-059 treatment	Reduce brain damage by scavenging excess free radicals such as ROS already produced by oxidative stress through neutralization	Did not show significant efficacy	Has been tested in clinical trials	Yang et al., 2021
Edaravone treatment	Reduce brain damage by scavenging excess free radicals such as ROS already produced by oxidative stress through neutralization	Recent clinical studies have shown that edaravone does not cause clinically significant QT prolongation as defined by the ICH E14 guidelines for the treatment of patients with cerebral hemorrhage, providing assurance of its safety	Has been tested in clinical trials	Nakamura et al., 2008
Combined treatment with N-acetylcysteine and selenium	Reduce brain damage by scavenging excess free radicals such as ROS already produced by oxidative stress through neutralization	Recent clinical studies have shown that this treatment modality slows the progression of perihematomal edema PHE in ICH patients and reduces the time to achieve the target RASS (Richmond Agitation Sedation Scale) and the length of stay in the ICU	Has been tested in clinical trials	Lyden et al., 2007
Nrf2 activator carotenoid therapy	Activation of the Nrf2 pathway to enhance antioxidant effects, thereby reducing brain damage from oxidative stress	Radiothione activates Nrf2 in ICH-affected brain tissue and reduces ICH-induced neutrophil counts, oxidative damage, and behavioral defects in animal studies	Has been tested in animal models	Wang et al., 2007; Zhao et al., 2007
Novel Nrf2 activator RS9 therapy	Activation of the Nrf2 pathway to enhance antioxidant effects, thereby reducing brain damage from oxidative stress	The results of the ICH mouse experiment suggest that the activation of Nrf2 by RS9 exerts a neuroprotective effect mediated by the attenuation of oxidative stress	Has been tested in animal models	Shimizu et al., 2021

#### Mitigating Brain Damage From Oxidative Stress by Preventing Excess Production of Free Radicals, Such as ROS, Immediately After ICH

Since the lysis of red blood cells after ICH produces hemoglobin, heme, and free radical substances, haematoma can be regarded as the source of excessive free radical production. Therefore, if blood clots is removed through surgical interventions, it should reduce oxidative damage. Minimally invasive surgery (MIS) for the evacuation of clots may demonstrate the feasibility of this idea, and related animal studies have found that the neurological function of experimental animals is improved through MIS treatment or when combined with other therapies. This provides good protection, as oxidative damage and cell apoptosis are correspondingly reduced (Wu et al., 2011; Liu et al., 2016; Wang et al., 2019). In the same way, as desferrioxamine mesylate (DFO) is an iron chelator, DFO can bind to excess iron produced after ICH, thereby reducing free radical production and oxidative damage. The therapeutic efficacy of DFO for ICH has been demonstrated in several preclinical studies (Okauchi et al., 2010; Hatakeyama et al., 2013; Xie et al., 2014). The tolerability, safety, and maximum tolerated dose of DFO in patients with ICH were determined in a phase I clinical trial (Selim et al., 2011). Preliminary results from a phase II trial showed that DFO reduced perihematomal edema (Yeatts et al., 2013). Meanwhile, some studies have found a positive prognostic effect of DFO use in patients after ICH, but more trials are needed (Zhao et al., 2015). The work of Zhu et al. (2021) suggests that the combined use of DFO and other scavengers may be a routine and more effective approach to treat oxidative damage after ICH. However, coverage of the CNS after ICH may be inadequate due to limitations possibly due to systemic toxicity associated with intravenous DFO, and no therapeutic effect was shown in the iDEF trial evaluating intravenous DFO after ICH (Yeatts et al., 2013; Selim et al., 2019). Therefore, the effect of DFO on ICH needs to be further investigated in depth. Additionally, a selenium nanocomposite material has been developed to reduce oxidative damage after ICH by preventing the cellular accumulation of ROS. The positive effect on oxidative damage shows its great potential for the clinical treatment of ICH and brain damage related to oxidative stress (Yang et al., 2021).

#### Neutralizing Excessive Free Radicals Produced by Oxidative Stress to Reduce Brain Injury

Neutralizing excess ROS and restoring the normal functions of endogenous antioxidant enzymes and scavengers is an effective way to eliminate excess free radicals. Many free radical scavengers have been evaluated in clinical trials. As a free radical scavenger, Nxy-059 showed good safety and tolerability in its ICH efficacy study, but unfortunately efficacy itself was not shown to be significant (Lyden et al., 2007). In the most recent study on ROS scavengers, the combination of N-acetylcysteine and selenium significantly slowed the progression of perihematomal edema in patients with ICH and reduced the time to reach the target Richmond Agitation Sedation Scale (RASS) and the length of stay in the ICU, although there was no significant change in neurological outcomes (Kim et al., 2021). As a free radical scavenger, edaravone has been used in Japan in patients with acute cerebral obstruction. Studies on its use in the therapy of ICH have been highly successful, and it has demonstrated significant neuroprotective effects in animal studies of ICH. In clinical trials, edaravone has also been shown to have better efficacy and safety for patients (Edaravone Acute Infarction Study Group, 2003; Nakamura et al., 2008; Shang et al., 2015; Shimizu et al., 2021).

#### Activation of the Nrf2 Pathway to Enhance Antioxidant Effects Aimed at Reducing Brain Damage From Oxidative Stress

Nrf2 is a key transcription factor for antioxidant response element (ARE) regulatory genes, which play an important regulatory role in cell survival. In oxidative stress after ICH, Nrf2 induces and up-regulates cytoprotective and antioxidant genes, attenuates tissue damage, and exhibits significant protective effects (Wang et al., 2007; Zhao et al., 2007). Radiothione is a natural isothiocyanate that induces the expression of several Nrf2-responsive genes. It is able to activate Nrf2 pathways and protect neurons from oxidative stress damage after ICH, manifesting a marked neurofunctional protective effect (Wang et al., 2007; Zhao et al., 2007). In a different study, Masomi-Bornwasser J and colleagues found that RS9, a novel Nrf2 activator, upregulated the expression of antioxidant enzymes such as SOD1 and HO-1 by activating the Akt-Nrf2 pathway. It also showed a protective effect on BBB and neuronal cells in the SBI of ICH (Sugiyama et al., 2018). It can be inferred that RS9 may be one of the effective therapeutic candidates for the treatment of SBI after ICH. The above experiments reflect that activation of the Nrf2 pathway is an effective antioxidant modality that significantly attenuates SBI produced by oxidative stress after ICH. This provides a new direction of thought for the treatment of ICH.

#### Other Antioxidant-Related Treatments

Hypothermia treatment is a known effective means of neuroprotection. According to Song's study, subhypothermia treatment in a rat model of ICH significantly achieved neuroprotective effects by inhibiting ICH-induced neuronal autophagy and apoptosis, reduced neutrophil infiltration and oxidative DNA damage (Song et al., 2018). Furthermore, clinical studies have shown that subhypothermia treatment for 8–10 days in patients with ICH can significantly reduce perihemorrhagic edema and decrease mortality (Staykov et al., 2013).

Mesenchymal stem cells (MSCs) are pluripotent cells with anti-inflammatory, antiapoptotic, and immunomodulatory properties (Nakano and Fujimiya, 2021). Numerous studies have shown that MSCs can reduce neurological deficits resulting from ICH (Zheng H. et al., 2018). The exosomes secreted by MSCs are considered the main mechanism of MSC therapy (Han et al., 2018). The release of exosomes has been shown to inhibit neuroinflammation and brain hemorrhage injury (Li et al., 2021), mainly through antiapoptotic, oxidative stress, and anti-inflammatory effects (Cai et al., 2020; Duan et al., 2020). Furthermore, Wang et al. evidenced the protective effect of umbilical cord MSC-derived exosomes on injured lateral hippocampal neurons after ICH (Wang et al., 2022). The long-term 5-year safety and possible beneficial effects of autologous MSCs transplantation have been clinically substantiated (Zheng H. et al., 2018). This is encouraging for the potential of MSC exosome therapy for application in the treatment of secondary injury after ICH.

## Conclusion

Oxidative stress is the main source of secondary injury after ICH. It is closely related to other processes, such as inflammation and autophagy. Therefore, the mechanism between oxidative stress and secondary injury after ICH should be further explored. After more in-depth research, it is hopeful that there will be a clearer trajectory for the clinical treatment of oxidative damage after ICH, in addition to contributing to the research and development of related drugs. More extensive literature on the topic may also inspire us to develop new clinical treatments for ICH.

## Author Contributions

LS and LM collected information and drafted and revised the manuscript. SC contributed to collecting information and editing the manuscript. LM directed the work and finalized the manuscript. All authors agreed to be accountable for the content of the work. All authors contributed to the article and approved the submitted version.

## Funding

This project was supported by Natural Science Foundation of Zhejiang Province (Grant no. LY21H090007) and Health Commission of Zhejiang Provincial Project (Project no: 2022510849).

## Conflict of Interest

The authors declare that the research was conducted in the absence of any commercial or financial relationships that could be construed as a potential conflict of interest. The reviewer SC declared a shared parent affiliation with the author(s) to the handling editor at the time of review.

## Publisher's Note

All claims expressed in this article are solely those of the authors and do not necessarily represent those of their affiliated organizations, or those of the publisher, the editors and the reviewers. Any product that may be evaluated in this article, or claim that may be made by its manufacturer, is not guaranteed or endorsed by the publisher.

## Abbreviations

ARE: Antioxidant response element
BBB: Blood–brain barrier
DFO: Desferrioxamine mesylate
HO: Heme oxygenase
ICH: Intracerebral hemorrhage
MPO: Myeloperoxidase
MSC: Mesenchymal stem cells
ROS: Reactive oxygen species
SBI: Secondary brain injury
SOD: Superoxide dismutase.
